# Circulating Virus Load Determines the Size of Bottlenecks in Viral Populations Progressing within a Host

**DOI:** 10.1371/journal.ppat.1003009

**Published:** 2012-11-01

**Authors:** Serafín Gutiérrez, Michel Yvon, Elodie Pirolles, Eliza Garzo, Alberto Fereres, Yannis Michalakis, Stéphane Blanc

**Affiliations:** 1 Unité Mixte de Recherche BGPI, INRA-CIRAD-SupAgro, TA A-54/K, Campus International de Baillarguet, Montpellier, France; 2 Unité Mixte de Recherche MIVEGEC 5290, CNRS-IRD-UM1-UM2, IRD, Montpellier, France; 3 CSIC-CCMA, C/Serrano 115 dpdo., Madrid, Spain; CSIC, Spain

## Abstract

For any organism, population size, and fluctuations thereof, are of primary importance in determining the forces driving its evolution. This is particularly true for viruses—rapidly evolving entities that form populations with transient and explosive expansions alternating with phases of migration, resulting in strong population bottlenecks and associated founder effects that increase genetic drift. A typical illustration of this pattern is the progression of viral disease within a eukaryotic host, where such demographic fluctuations are a key factor in the emergence of new variants with altered virulence. Viruses initiate replication in one or only a few infection foci, then move through the vasculature to seed secondary infection sites and so invade distant organs and tissues. Founder effects during this within-host colonization might depend on the concentration of infectious units accumulating and circulating in the vasculature, as this represents the infection dose reaching new organs or “territories”. Surprisingly, whether or not the easily measurable circulating (plasma) virus load directly drives the size of population bottlenecks during host colonization has not been documented in animal viruses, while in plants the virus load within the sap has never been estimated. Here, we address this important question by monitoring both the virus concentration flowing in host plant sap, and the number of viral genomes founding the population in each successive new leaf. Our results clearly indicate that the concentration of circulating viruses directly determines the size of bottlenecks, which hence controls founder effects and effective population size during disease progression within a host.

## Author Summary

Infecting viruses progress within multi-cellular hosts via two distinct mechanisms: cell-to-cell proximal contamination and long-distance migration to remote organs through the vasculature. In distant susceptible organs, it seems logical that the number of initially infected cells, the number of viral genomes entering each of these cells, and thus the number of founders of new viral “colonies”, depends on the concentration of infectious units transported in the vasculature. For any organism, the number of founders colonizing a “virgin territory”, is of prime importance in determining the forces driving its evolution. This is particularly true for viruses where the so-called founder effect is a key factor in the emergence of new variants with altered virulence. It is surprising to note, however, that whether the circulating virus load directly drives the size of viral populations during host colonization remains elusive. By monitoring for the first time the virus concentration flowing in host plant sap, in parallel with the number of viral genome founders in each successive leaf, we provide unequivocal evidence that the concentration of circulating viruses can directly determine the founder effect and effective population size during disease progression in a eucaryotic host.

## Introduction

Virus progression within multi-cellular hosts operates via two distinct mechanisms: cell-to-cell proximal contamination and long-distance migration (either as free infectious units or in circulating cells) to colonize new organs and/or tissues. Both animals and plants can be considered as heterogeneous landscapes consisting of an ensemble of very different organs or “territories”, variably distant, and interconnected by a complex vascular system transferring nutrients, metabolic product and wastes, and information. Soon after entry into a healthy host, the vast majority of viruses use this connecting vasculature to travel long distances and expand their populations into virgin territories. In distant susceptible organs, it seems intuitively obvious that the number of initially infected cells, the number of viral genomes entering each of these cells, and thus the number of founders of new viral “colonies”, will depend on the concentration of infectious units transported in the plasma or sap flooding the vasculature. In other words, the virus load in the circulating flux will determine the bottlenecks in a virus population progressing within a host, and hence the effective population size and the pace at which new variants are produced, selected, and emerge. Taking an alternative view, however, one could speculate that the number of “entry points” into various organs of the host might sometimes be extremely limited, and hence the number of founder viral genomes in such territories may always be low or constant, regardless of the circulating virus load. Surprisingly, for both animal and plant viruses, experimental data supporting one or the other of these contrasting scenarios are extremely rare and only fragmentary at best.

For animal viruses, many studies have quantified the virus load in the plasma of infected individuals. That this virus load changes drastically during progression of the infection, upon the onset of host defenses or during drug treatments, has been reported for several viruses, e.g. hepatitis B virus (HBV, [1]), hepatitis C virus (HCV, [2], [3]), human immunodeficiency virus (HIV, [2], [4], [5]), simian immunodeficiency virus (SIV, [6]), and poliovirus [7]. The logical speculations that an increase in plasma virus load increases the infection dose in various tissues of the host [1], [6], [8], in some cases allowing viral access to specific organs [7], [9]–[12], enlarges the viral population size and most likely augments the number of multiply infected cells, thus favoring recombination [13]–[16], have been discussed frequently. Interestingly, a very recent study demonstrated an ongoing exchange of HIV genomes between the plasma and CD4(+) T blood cells [17]. However, despite its recognition as a priority question [16], this expected relationship between circulating virus load, effective viral population size, and the multiplicity of cellular infection (MOI) remains to be characterized.

In plants, this question has thus far been totally hampered by long-standing technical difficulties with the collection and analysis of pure phloem sap for determining virus titer. Collection of phloem sap exuding from sieve tubes in the veins of severed or wounded stems works in only a few plant species [18], and the wounded cells surrounding the sieve tubes always contain viruses that can contaminate the exudates. A very sophisticated alternative technique uses aphid stylets as pure-phloem-sap collecting tools [19], but its implementation is laborious in practice and produces only minute amounts of sap per treated insect. As a result of these technical limitations, despite the fact that all plant viruses travel long distances within their host together with the elaborated sap, the viral titer (and its putative dynamic fluctuations) circulating within sieve tubes remains a complete mystery. Recently, we reported monitoring of within-host MOI in successive leaves of a host plant infected by *Cauliflower mosaic virus* (CaMV, [20]). We demonstrated that the MOI varies greatly among leaf levels, increasing as the infection progresses and later decreasing before flowering and senescence of the host. Although this variation could not be formally explained, we speculated that it could be driven by changes in the virus titer circulating within the plant vasculature. Thus, more viral infectious units may enter each leaf, and each individual cell within these leaves, as the titer in the sieve tubes increases.

In addition to an augmented MOI, this would translate into larger effective sizes of within-host viral populations, due to larger numbers of founder genomes in each leaf, and thus into mild (or no) bottlenecks at leaf entry. The viral population bottlenecks associated with leaf colonization have been investigated in several plant virus genera—Potyvirus [21], Tobamovirus [22], Cucumovirus [23], [24], and Caulimovirus [25]—but the bottlenecks detected were either not quantified [23], [24], quantified only at the single leaf level [22], or calculated as a single averaged value over the whole systemically infected host plant [21], [25]. It is interesting to note that physical host barriers [21]–[23], [25], [26] have often been speculated to induce bottlenecks at leaf entry. Most interestingly, however, a recent report is demonstrating that the viral dose could also be a major factor, thus convincingly concluding that it should be considered in future studies on within host population bottlenecks [27].

Here, we report the first quantification of a plant virus titer within the vasculature of its host, and monitoring of this important trait as infection progresses. In addition, we demonstrate that changes in sap virus titer correlate with changes in the size of viral population bottlenecks at leaf entry, strongly suggesting that the circulating virus load is a major factor determining the effective size of within-host viral populations.

## Results

### Monitoring of CaMV titer within the sieve tubes of infected turnip host plants

We first conducted a pilot experiment to collect pure phloem sap from severed aphid stylets inserted precisely within sieve tubes, as previously described [19], [28]. Although the considerable technical problems encountered led us to conclude that it was unreasonable to use this technique for further time-course experiments (for details see online Text S1), we could compile measures from 19 sap samples collected from different leaves at variable stages of development, and obtain an average value of 318 (SD +/−244) viral genomes per nanolitre of sap. The implications of this order of magnitude, i.e. tens to hundreds of CaMV virions per nanolitre, are discussed further below.

**Figure 1:**
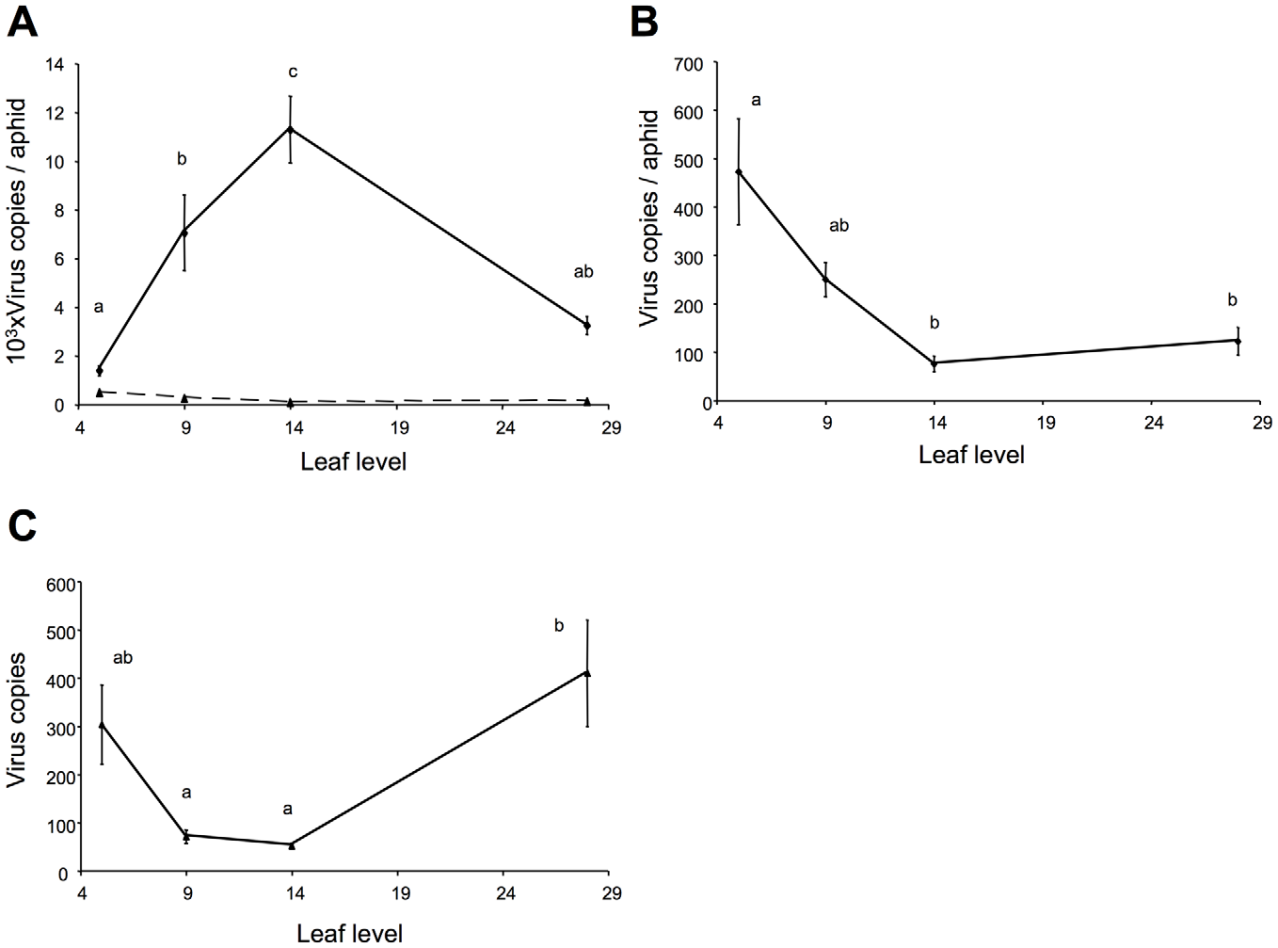
Dynamics of CaMV load in phloem sap. (A) Comparison of the mean virus load in aphids fed for 10 minutes (dashed line, see also B) and 16 hours (solid line) on the leaf levels indicated (vertical bars indicate standard error). The experiment was conducted in parallel on 20 plants. The difference between the two treatments was highly significant (linear mixed-effects model with leaf level and treatment as fixed effects and plant as random effect; p-value <0.001). Significant differences were also found among viral sap loads in successive leaf levels when aphids were fed for 16 hours (Tukey's HSD, different letters mean p-values <0.02). Results from aphids fed for 10 min. are further considered in B. (B) This panel shows the same data as in (A), for viral sap load in aphids fed for 10 minutes, but the scale of the y-axis enlarged 20 times (Tukey's HSD, different letters mean p-values <0.001). (C) Virus concentration in plant cells of the same leaf and leaf levels as in (A) and (B), expressed as normalized mean virus copies per actin gene copy. Although significant differences are observed among certain leaf levels, the average cellular virus load appears relatively constant, the range falling within the same order of magnitude (Tukey's HSD, different letters mean p-values <0.03).

We next decided to estimate the virus load directly within whole aphids, processed after a 16-hour acquisition period on CaMV-infected turnip plants—a time at which aphids are most often engaged in a phase of sustained sap ingestion from the phloem sieve tubes [29]. As discussed further below, a continuous sap flux transits rapidly within the aphid gut during this sustained phloem-feeding phase, and ingested CaMV is thought to simply follow this flux from ingestion to excretion without entering and accumulating within the aphid body [30]. We also carefully targeted young sink leaves to make sure that we were indeed analyzing virus load in aphids containing phloem sap flowing into the newly developing leaves (see Materials and Methods for a more detailed explanation). Using this technique, we monitored virus load in the sieve tubes at several successive leaf levels appearing on 20 plants infected in parallel, and were able to demonstrate huge temporal variations in the circulating virus titer. We measured an average of 1386 viral copies per aphid in the first systemically infected leaf level (level 5), with a sharp increase in leaf levels 9 and 14 before reaching a maximum of 11291 viral copies/aphid, and then a decrease back to initial values in leaf level 28 (Figure 1A). The differences observed between successive leaf levels proved highly significant (linear mixed-effects model, P<0.001).

The control experiment gave an estimate of what aphids can potentially acquire when feeding superficially only in epidermal and mesophyll cells. Because aphids always conduct test probes in these tissues before settling in the sieve tubes [28], we needed to assess the number of CaMV copies acquired during these test probes and how much this could affect our measurements of virus load in the sap. Aphids of the species *Myzus persicae* seldom reach deep phloem tissues before a feeding time of about 30 min has elapsed [29]. We thus performed exactly the same experiment in parallel, on the same plants and the same leaves, but allowing only a short feeding period of 10 min. Figure 1A shows that the virus load within aphids that have fed only in epidermal and mesophyll cells (dashed line) is much lower, and can be neglected when estimating the virus load from aphids fed in sieve tubes (solid line). By enlarging the scale (Figure 1B), it can be seen that the time-pattern of virus copy number in aphids fed only for a short time is distinctly different from that in aphids fed for longer, confirming that “long-fed” aphids indeed access a different tissue (sieve tubes).

Finally, we also immediately estimated the virus load in cells of leaves used directly for aphid-feeding experiments (Figure 1C). Interestingly, the range of CaMV accumulation among successive leaf levels was of the same order of magnitude (from ∼10 to ∼40, Figure 1C), suggesting that the efficiency of viral replication in these cells is rather constant. The time-pattern of virus accumulation in cells of successive leaf levels (Figure 1C) appeared totally different from that in the sap (Figure 1A), highlighting the absence of correlation of the dynamics of the virus load in these two distinct compartments (i.e. sieve tubes vs. mesophyll). Consistently, the difference in virus load in the sap between two leaf levels was not correlated to the corresponding difference in the mesophyll (Pearson r values were equal or inferior to 0.417, and p-values equal or superior to 0.067).

### CaMV population bottleneck size at entry to different leaf levels

**Figure 2:**
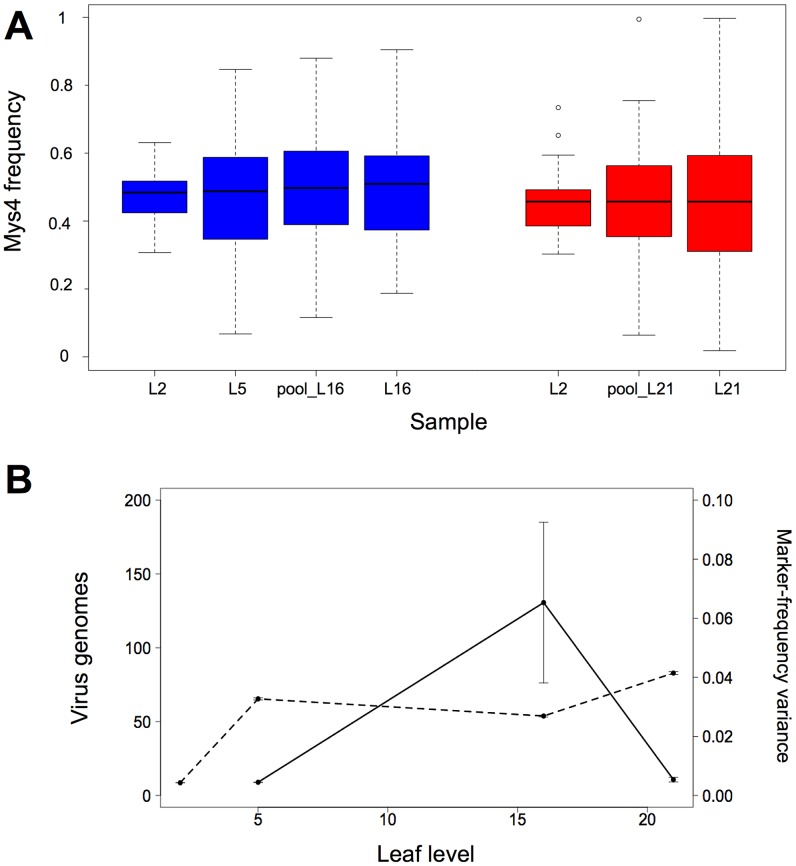
Bottleneck sizes at CaMV entry into different leaf levels. (A) Boxplot of the Mys4 frequency distributions in samples harvested successively in two sets of 50 replicate plants. In the first set of plants (blue), harvested samples were leaf level 2 (L2), leaf level 5 (L5), pooled leaves below leaf level 16 (pool_L16), and leaf level 16 (L16). In the second set of plants (red), harvested samples were leaf level 2 (L2), pooled leaves below leaf level 21 (pool_L21), and leaf level 21 (L21). The average frequency of Mys4 did not vary during CaMV progression into host plants, demonstrating the equi-competitiveness of Mys4 and Mys7 (linear mixed-effects model with leaf level as a fixed effect and plant as a random effect: p-values = 0.450/0.818, and the slope of the two linear regressions of Mys4 frequency over time is not significantly different from 0: p-values = 0.124/0.616). (B) The full line shows changes in the average number of CaMV genomes founding the population in successive leaf levels. The number of viral genome founders (CI_95%_) in leaf levels 5, 16 and 21 was: 8.8 (6.4–14.1), 124.7 (19.1–908.9) and 10.8 (7.1–23.9), respectively. The dashed line represents changes in the variance of Mys4 frequency among repeated plants at successive leaf levels (scale on the right). Confidence limits for variance values were obtained using a resampling technique (1000 bootstraps sampling 50 frequency values with replacement). Error bars represent standard errors.

We next assessed whether a higher virus concentration in the circulating sap represents a higher viral dose, which would in turn increase the number of founder viral genomes in newly infected leaves. For this experiment, we conducted a similar longitudinal analysis of infected plants, measuring the size of bottlenecks imposed on CaMV populations at the entry to successive leaf levels. Fifty replicate plants were inoculated with a mixture of two CaMV variants (Mys4 and Mys7) at a 1∶1 ratio, and their relative frequency was monitored in order to evaluate bottleneck sizes upon entry to leaf levels 5, 16 and 21, as described in the Materials and Methods. Briefly, Mys4 and Mys7 are equally competitive in doubly infected plants (Figure 2A) and the procedure consisted of comparing the variance in the relative frequency of Mys4/Mys7 among the 50 replicate plants, at an “initial” stage (source population) and at a “final” stage (population in a specific leaf level). Changes in variance during passage from the initial to the final stage were used to calculate the number of CaMV genomes from the source populations that made it through to each respective leaf level (according to [25]). As leaf level 5 was the first systemically infected level, we considered the corresponding source population to be that present in inoculated leaf level 2, for reasons described previously [22]. Thus, by analyzing the viral populations in leaves 2 and 5 in each of the 50 replicate plants, we were able to estimate the size of the bottleneck through which the CaMV population passes when leaving the inoculated leaf to initiate systemic infection. This protocol is not destructive since the inoculated leaves are collected just before senescence, and leaves at level 5 are punctured only to extract few leaf discs.

In contrast, the initial source populations from which viral genomes in leaf levels 16 and 21 originate are much harder to define because many leaves below them are exporting virions. We made the same assumption as in [25] and considered that, due to anastomosis of the vasculature connecting different leaves, all viral genomes present in all leaves below leaf level 16 (or 21) provide the best estimate of the source population feeding into this leaf level. Because in this case the protocol is destructive (most infected leaves are finally harvested), we could not analyze levels 5, 16 and 21 with the same set of plants. We thus estimated the bottleneck at entry of leaf levels 5 and 16 with one set of 50 plants, and that at entry of leaf level 21 with a second similar set under the same experimental conditions. The results, summarized in Figure 2B, illustrate the remarkable variation in bottleneck size at the entry to different leaf levels. While the number of founder viral genomes is very low in the first systemically infected leaf (around ten per leaf), it rises by over an order of magnitude in leaf-level 16, before decreasing back to initial values in leaf level 21.

### Dynamics of bottleneck size changes during host invasion

**Figure 3:**
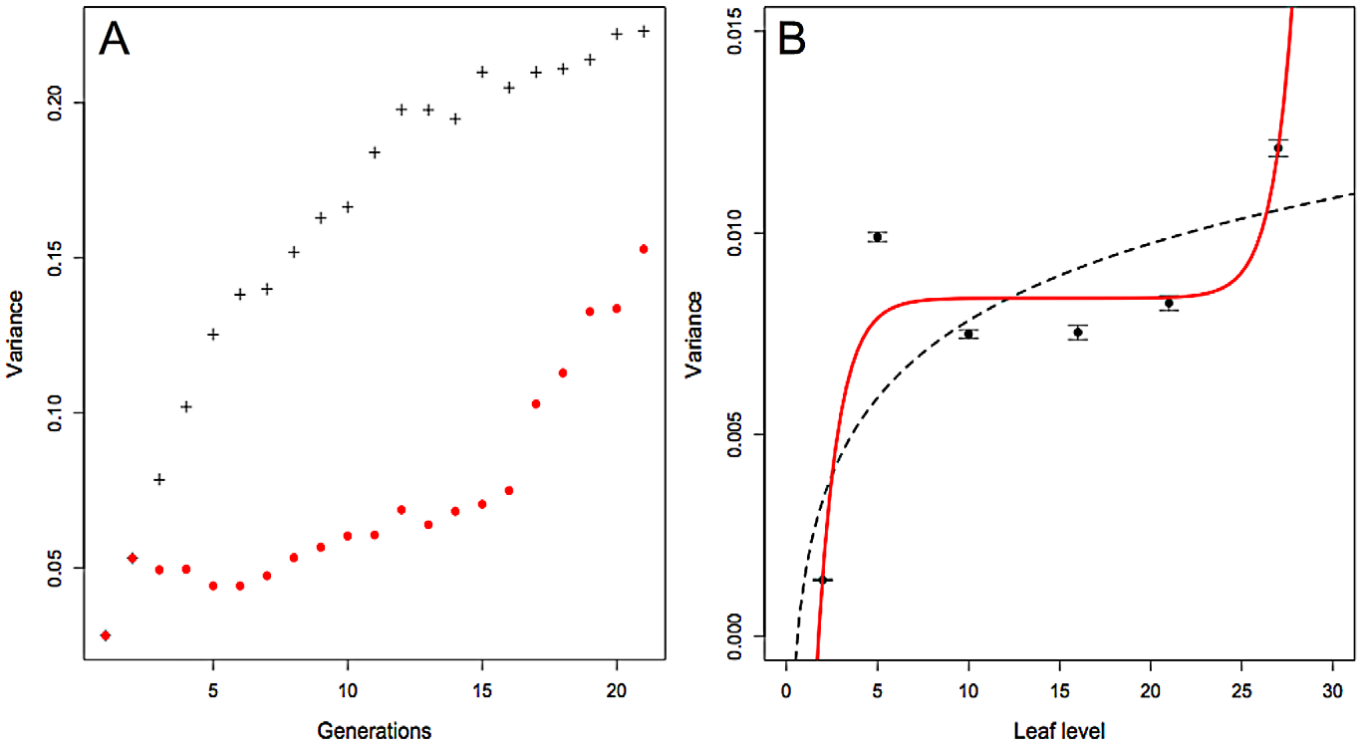
Monitoring of the bottleneck size changes during disease progression. **A**. Among-populations variance of allele frequency of a diallelic neutral locus over time when the effective size of each population is (i) always equal to 10 (black crosses); (ii) equal to 10 until generation 2, then equal to 100 until generation 15, and then equal to 10 again (red circles). All populations start with the same initial allele frequency of 0.5. **B.** At each indicated leaf level, the variance of Mys4 frequency was estimated among 40 replicate plants. The equi-competitiveness of Mys4 and Mys7 was again confirmed in this experiment, by showing that the linear regression of Mys4/Mys7 relative frequency over time is not different from 0 (p-value = 0.053; not shown). Confidence limits for variance values were obtained as in Figure 2B. Error bars indicate standard errors. Two functions were fitted to the variance values obtained over time. Red line: a hyperbolic-sinus function with the formula: y = d*(b/d+sinh(c*(x−a))); where a = 14.84, b = 8.37E-03, c = 0.87 and d = 2.28E-07; this function has an AIC = −54504.68. Dashed line: a logarithmic function with the formula: y = a+b*ln(x); where a = 1.45E-3 and b = 2.764E-3; this function has an AIC = −52021.35.

To confirm the observed changes in bottleneck size over time (Figure 2B), we argued that one could simply track the dynamics of variance in the relative frequency of Mys4/Mys7 among replicate plants at successive leaf levels. As Figure 3A illustrates, if the within-plant effective population size were constant the variance of the marker frequencies between plants would increase monotonically. On the other hand, temporal variations of the within-plant population size are expected to induce a non-monotonic behavior of the among-plant variance. For example, if the population size is first small, then large and then small again, the among-plant variance in marker frequency is expected to first increase rapidly, then increase very slowly and then resume a rapid increase. Thus, simply tracking the between-plant variance, without the need to identify and analyze the initial source populations entering into each leaf level, should allow us to infer the pattern of bottleneck size changes with time, although it cannot allow actual estimation of bottleneck size *per se*.

We first plotted the variances of marker frequency calculated from the two sets of plants used in Figure 2B (dashed line), and verified that it increased when the bottleneck at leaf entry was narrow, whereas it remained approximately constant when the bottleneck was relaxed. Then, we decided to confirm the pattern of bottleneck size changes over time on a new single set of 40 Mys4/Mys7-infected plants. We harvested leaf levels 5, 10, 16, 21 and 27, leaving all other leaves intact, thus allowing the plants and the infection to grow continuously and develop throughout the experiment. Consistently, Figure 3B shows that the among-plant variance of Mys4 relative frequency increases between leaf levels 2 and 5, stabilizes in leaf levels 10 and 15, and again increases in leaf levels 21 and 27. We verified that the results were better explained by a non-monotonic function, such as a hyperbolic-sinus function, as opposed to a monotonic function, such as a logarithmic function, by comparing their Akaike's Information Criterion (AIC) values. The best model (lowest AIC) was the hyperbolic-sinus function (ΔAIC_hyperbolic-logarithmic_>2000), confirming the pattern of bottleneck changes shown in Figure 2B.

Remarkably, the pattern of bottleneck size changes at successive leaf levels (Figures 2 and 3) resembles that of the virus load measured in sieve tubes of corresponding leaf levels (Figure 1), strongly suggesting a correlation between the two.

### Relationship between virus sap-load and bottleneck size at leaf entry

Though stemming from different plant sets, the above results together suggest a match between the pattern of the virus concentration within the sap and that of the size of the bottlenecks endured by CaMV populations when initially colonizing leaves. In order to confirm this relationship in a single experiment, we further analyzed DNA samples used in Figure 1. As indicated in the Materials and Methods, the plants were infected by a mixture of the two CaMV genotypes (Mys4/Mys7 ratio 1∶1), allowing the monitoring of the variance of this ratio both within aphids (so within the phloem sap) and within the corresponding leaf tissues, thus allowing to calculate the number of viral genomes passing from the sap into the leaf tissues. The data summarized in Figure S1 (in Text S1) consistently confirm an increase of the number of viral colonizers within leaves, when the viral load increases in the sap. Early and late in infection, when viral sap-load is minimum, the size of CaMV population bottlenecks is close to 10, whereas it rises above 70 as viral sap-load drastically increases. The sizes of CaMV populations colonizing leaves were very similar to those calculated in the other set of plants in Figure 2B.

## Discussion

### Virus load in phloem sap

Unlike the case in mammals, the virus load in the vasculature of plants represents hard-to-access information. There is no reported method of artificially puncturing sieve tubes and aspiring sap; simply severing the stem is, in most cases, totally inefficient as in many plant species (including turnip as used here) no liquid exudes from the wound [18]. In addition, the viral content of companion and other cell types that always surround the sieve tubes represents an inevitable source of putative contamination upon wounding. For these reasons, and because they are natural entities capable of pumping pure sap, in this study we attempted to exploit aphids to access this plant compartment. Because of the multiple drawbacks encountered by us (see Text S1) and others [19], the sophisticated stylectomy technique was implemented only in an exploratory experiment, not differentiating leaf level and leaf age. How the resulting range of tens to hundreds of CaMV genome copies per nanoliter (average^+/−SD^ 318^+/−244^ copies/nl) compares with values quantified from whole aphids (Figure 1A) depends on the volume of sap that an aphid actually contains, and whether or not virions are massively degraded in the aphid gut. It can be calculated from [31] that aphids of the species *Myzus persicae* (Sulz.) can individually contain around 25 nl of sap, continuously flowing and rapidly transiting (about 1 hour) from ingestion to excretion. This volume would consistently correspond to a range of 55–452 virus copies per nanoliter of sap, depending on the leaf level (number of copies estimated from pools of five aphids, divided by 5 to provide a per aphid number as in Figure 1, and by 25 to provide a per nanoliter number). The current literature suggests that aphids (and other hemipteran insects) readily assimilate free amino acids, but poorly degrade proteins [32—34] [35]. However, in additional experiments where aphids were fed with artificial solutions of known virus concentrations, a similar calculation of the virus load in aphids (as above) gave values about one order of magnitude lower than that measured in the feeding solutions (Figure S2 in Text S1). As detailed in the section 6 of the Text S1, despite clear evidence that aphids feed much less on artificial membranes than on plant leaves (Figure S3 and Table S5, in Text S1), we cannot exclude a possible degradation of some virions within the gut. All caution considered, we thus propose the above values (55–452 virus copies/nl) as a conservative estimate of the average CaMV sap load in turnip hosts. In any case, would aphids partly decrease the number of detectable viral copies per nanoliter of sap, they would most likely equally do so for all leaves tested, letting the dynamic pattern in Figure 1 unaffected.

The approximation reported here for the viral sap load is certainly specific to CaMV infecting turnip; however, it represents the first estimation of the load of a plant virus circulating within the vasculature of its host. In addition to the cautionary remark mentioned above, to reliably reflect the virus load in plant sap, the technique used in Figure 1 requires the use of an insect species specifically feeding in sieve tubes, and in which the virus does not accumulate. This condition is fulfilled in the case of aphids and non-circulative viruses (like CaMV), because only a few viral particles are specifically retained within the anterior part of the feeding apparatus [36]–[39] (see also dashed line in Figure 1A). Circulative viruses appear more problematic, as they can penetrate the body of their vectors and accumulate, or even replicate, in gut cells, salivary gland cells, or elsewhere [40]. We propose that by using phloem-feeding insect species that are non-vector, and by verifying that they do not accumulate the studied viruses, this technique can be transferred to virtually all plant virus species.

It is generally and intuitively assumed that the number of viral particles circulating within the vasculature depends on the replication rate in the cells that shed virions into the plasma or sap, and on the number of such infected cells within the host [6], [41], [42]. Accordingly, early studies quantifying the accumulation of *Tobacco mosaic virus* in different leaves (reviewed in [43]) speculated that changes in within-leaf accumulation might correlate with changes in sap load. However, because we found small differences in CaMV accumulation at comparable development stages of successive leaf levels (cf Figure 1A and 1C), and because the CaMV load in the mesophyll does not correlate with that in the sieve tubes, we believe that changes in the CaMV replication rate are not responsible for the variations in the sap observed here. Rather, it is the fact that more cells and leaves are becoming systemically infected, and thus cumulatively shedding virions within the sieve tubes, that accounts primarily for the increase in viral sap load, as previously speculated [20]. In contrast, the drop in virus load late in infection is surprising and invites speculation on several hitherto unreported aspects of plant virus biology, including a possible arrest of virion export from infected leaves, an increased rate of virion degradation within the sap, or massive and rapid storage in unknown plant compartments (for example roots) clearing the vascular system. For animal viruses, virion turnover in plasma results from the constant production by infected cells, and rapid degradation due to intrinsic instability and attack by the immune system. The half-life of circulating virions—studied for HCV, HIV and HBV [1], [2]—has been demonstrated to be extremely short, in the range of one to a few hours. Equivalent studies do not exist in plants, and there is no mention of any possible virion decay and/or turnover within the sap in the available literature. Although CaMV produces very stable virus particles, as demonstrated by purification procedures involving 1.5 M Urea, 2% Triton, 1 CMC β-OG and butanol treatments [44], our results suggest that they might be degraded in (or removed from) the sap with an unknown half-life time. Whether the leaves stop exporting virions at some point, or whether unknown mechanisms act to increase degradation, or to sequester virions in an as yet undetermined compartment, is unclear, but such questions set an interesting scene for future investigations.

### Virus load in sap drives bottleneck sizes

Variation in the size of the bottlenecks undergone by CaMV populations at leaf entry has been analyzed here with previously described procedures. Entry of leaf level 5 was analyzed using a protocol similar to that described by Sacristan and co-workers [22], which identified precisely the initial (within leaf 2) and final (within leaf 5) populations. In contrast, when quantifying the CaMV genome founders colonizing leaf levels 16 and 21, the originating population is elusive and has been suggested to be best illustrated by the overall population present within the plant [25]. Because the two different protocols used in these previous studies have been suspected to differentially affect the outcome of the experiments [45], we developed a third and novel approach (Figure 3), allowing a similar analysis at all leaf levels in a single set of plants. In this approach, approximately one leaf level out of five is harvested, thus plant development is affected only minimally. While the sizes of the bottlenecks cannot be estimated in this way due to a lack of information on the source population colonizing each individual leaf, the pattern of dynamic changes in bottlenecks can be tracked efficiently together with the spread of infection. This additional experiment demonstrated that bottlenecks are consistently narrow at early and late time points in infection, and relaxed at intermediate stages, thus signifying that the two distinct protocols previously used for bottleneck quantification do not bias the results. Interestingly, the viral population within the sap flowing into leaves is the real source population, and we could use it to calculate and confirm the bottleneck sizes at leaf entry (Figure S1, in Text S1). Though in this experiment, the leaves were at early stages of development (still sink), the estimated number of viral colonizers was remarkably similar to that established from fully matured leaves (compare Figure 2B and Figure S1 in Text S1).

The different sets of plants analyzed indicate that the dynamic pattern of virus load within the sap is very similar to that of bottleneck size at the entry to successive leaf levels. This strongly suggests a direct relationship between the two, and we propose that (i) in the first systemically infected leaf, the virus titer in the sap is far too low to saturate all existing entry points, and the limiting factor is thus the availability of virions; (ii) as more leaves become infected and shed virions within the sap, the virus titer increases and relaxes the bottleneck during disease progression, with no apparent limit due to entry points; and finally (iii) the situation reverts later in infection by unknown mechanisms that either halt virus export from infected cells, store virions elsewhere than in the vasculature, or accelerate virion decay in the sap (or a combination thereof), resulting in a drop in the number of circulating virions and thus of the size of bottlenecks. Interestingly, we previously described a very similar pattern of dynamic changes in the MOI of CaMV at different leaf levels, indicating that the CaMV load in the sap probably influences the number of viral genomes entering leaves as well as individual cells within the leaves [20].

In the scenario suggested here for CaMV, the most significant bottleneck encountered during the virus life cycle is certainly that imposed by aphid transmission, during which very few genomes might be inoculated, as suggested by studies with other non-circulative viruses [36], [38], [46]. The infection would then evolve with an increasing population size limited only by the number of virions produced and loaded into the vascular system. Whether this scenario holds true for other plant viruses is totally unknown. Interestingly, the procedure developed here to study in parallel the fluctuation of virus sap-load and the number of founders entering each leaf is certainly transferable and may inform on this question.

## Materials and Methods

### Engineering genetic markers into full-length CaMV clones

The genome of CaMV (genus *Caulimovirus*) is a circular dsDNA of approximately 8000 bp (depending on the strain), encoding seven independently translated open reading frames (ORF I-VII) [47]. The QuickChange Site-Directed Mutagenesis kit (Stratagene) was used to insert 39-bp oligonucleotides between ORF I and ORF II of plasmid pW260 [48], to be used as genetic markers as previously described [49]. Two distinct clones were engineered with this technique (primer sequences available upon request), containing 39 additional nucleotides (TCTACATATTCCTGATAACTCAACGGTCGTCGACGGAGT or AGTAAGTGCTGTAAGTATAATAAGGATACTTGTCGACAG) between the stop codon of ORF I and the start codon of ORF II. These two clones were named Mys4 and Mys7, respectively, and a real-time PCR protocol was developed for their specific quantification in DNA mixtures. PCR conditions and primers are detailed in the online supporting information (Text S1). Clones Mys4 and Mys7 were tested for infectivity, and the stability of the introduced genetic markers was confirmed by sequencing viral populations after two serial passages of 21 days each in turnip host plants. The symptoms induced by both Mys4 and Mys7 were similar to those induced by the parental pW260 clone. Virus particles were purified from infected plants, quantified and stored as previously described [49], [50].

### Aphid rearing, plant growth conditions, inoculation and sampling

Colonies of the aphid species *Myzus persicae* were maintained in insect-proof cages on eggplant, in a growth chamber at a temperature of 23/18°C and a photoperiod of 14/10 h (day/night), conditions ensuring that the colonies are reproducing clonally. The colonies were transferred to a new cage and new host plants every two weeks, and aphids were always collected at the moment of the transfer, thus at comparable population densities. Turnip plants (*Brassica rapa* cv. “Just Right”) were maintained in an insect-proof growth chamber under controlled conditions (temperature 24/15°C, photoperiod 15/9 hours day/night). For all experiments, plantlets at the third leaf stage were inoculated mechanically by rubbing a virus suspension containing 400 ng of virus particles and Carborundum abrasive powder, on the second leaf level. All inoculums were prepared by mixing Mys4 and Mys7 purified virus particles at a 1∶1 ratio. The appearance of new leaves (budding) on the inoculated plants was monitored and noted daily in order to allow leaf sampling at a precise leaf age. Depending on the experiment, either entire leaves or six leaf discs (0.8 cm ø) distributed evenly over the leaf surface were sampled. DNA from each leaf sample was extracted as described [51].

### Collection of sap from sieve tubes

Twenty replicate plants were inoculated and sampled at four time points. The different time points corresponded to leaf levels 5, 9, 14 and 28. The first systemically infected leaves appeared at leaf level 5, the first visual signs of flowering induction (changes in leaf morphology) appeared irregularly between leaf levels 21 and 28, and the plants slowly entered senescence after leaf level 28. We originally planned to collect also leaf level 21, but our aphid colony unfortunately collapsed at this time and recovered only in time to use leaf level 28. Because of the length of the experiment, different aphid cohorts were used at different dates for different leaf levels, but they all originated from the same clonal rearing, maintained in constant conditions and collected at comparable population densities. It is important to note that the sampling dates were chosen when leaves were in their 5^th^ day of development. At this stage, the sink-to-source transition has not yet occurred and the phloem sap flows into the developing leaves, whereas this flow is inverted after the transition that occurs on approximately the 10^th^ day of leaf development, when each leaf exceeds 1/3 of its final size [52].

For each time point, fifty aphids were confined in a “cage” enclosing the defined leaf level (see above) on each of 20 replicate plants. Three groups of five immobile aphids—those most likely feeding—were collected from each leaf after an acquisition period of 10 minutes in epidermal and mesophyll cells [29], and instantly frozen in liquid nitrogen. The remaining aphids were caged again and left on the leaves overnight (16 hours). After this overnight period, three groups of five immobile aphids were collected from each leaf and similarly frozen in liquid nitrogen. *M. persicae* generally settle and feed continuously within the sieve tubes during such a long period [29], and we thus assumed that most of them would contain sap from which the CaMV genome copy number could be quantified. As CaMV is transmitted in a non-circulative manner, it does not accumulate within the vector body and is believed simply to follow the sap flow in the aphid's gut, from ingestion in the stylets to the excretion of honeydew [30]. Finally, all remaining aphids were discarded and the entire corresponding leaves were immediately collected and stored at −20°C until use. The DNA from each group of 5 aphids was extracted as previously described [53] and analysed by Q-PCR. The total DNA from each leaf was also extracted as described [51], and analyzed by Q-PCR. The validation of this approach by feeding aphids with artificial suspensions with known virus concentrations is presented in the point 6 of the Text S1.

### Estimating the size of the CaMV population bottleneck at leaf entry

We estimated the size of the CaMV population bottlenecks during colonization of leaf levels 5, 16 and 21, which appear successively on infected plants. In this experiment, leaves were collected on the 13^th^ day of development, after the sink-to-source transition, when colonization by viruses imported from the phloem sap is over [52].

Two sets of 50 plants were inoculated with a mixture of Mys4 and Mys7 purified virions, on leaf level 2. In both sets, we collected the inoculated leaves just before their death in order to minimally affect (if at all) virus exit and migration towards systemically infected leaves. In addition, in one of the sets of plants, we sampled leaf discs on leaf level 5 and let the plants grow until leaf level 16 appeared. Using an approach similar to [25], we then collected a leaf pool containing all leaves except leaf 16, which was left to develop for 13 days and finally harvested. The second set of plants was treated similarly for analysis at leaf level 21, collecting pooled leaves below nascent leaf 21, which was finally collected 13 days later.

DNA was extracted from all leaf samples, and the relative frequency of Mys4/Mys7 was estimated using Q-PCR as indicated above. In the first plant set, we determined the variance of Mys4/Mys7 relative frequency among leaves 2 (a), among leaves 5 (b), among pools of leaves below leaf 16 (c), and among leaves 16 (d). In the second plant set, we determined the variance of Mys4/Mys7 relative frequency among pools of leaves below leaf 21 (e), and among leaves 21 (f). Comparing a–b, c–d, and e–f allowed evaluation of the size of the bottlenecks at the entry of leaf levels 5, 16 and 21, respectively, as described [25].

### Alternative method to reveal the pattern of bottleneck changes during plant invasion

In order to rapidly establish the pattern of bottleneck changes in a viral population invading successive leaf levels, we tracked the among-plant variance in relative marker frequency over time. Severe bottlenecks should increase among-plant variance, while mild bottlenecks should affect it only slightly. We expected that if the viral effective population size was constant throughout the infection the among-plant variance would increase monotonically, while temporal variation in the effective size would induce non-monotonicity in the pattern of variation of among-plant variance. To illustrate the patterns that could arise from such temporal variation in the effective population size, we simulated the evolution of a single diallelic neutral locus in a number of unconnected populations, representing the different host plants. All populations had the same effective size at any given time, but their effective size could vary over time. We computed the among-population variance in allele frequencies over time. The results, e.g. Figure 3A, confirmed our expectation: when the populations evolve under constant effective size, the among-populations variance increases monotonically. When the effective population size varies over time, the increase in among-populations variance is fast when the effective population size is small and slow when it is large.

To experimentally investigate how among-plant variance in relative marker frequency evolved over time, 40 plants were inoculated in parallel and sampled at six different time points, corresponding to leaf levels 2 (inoculated leaf), 5, 10,16, 21 and 27. Each leaf level (except leaf level 2, which was collected just before death) was sampled when leaves were in their 13^th^ day of development by collecting the entire leaf. The Mys4/Mys7 relative frequency was estimated (by Q-PCR as above) in successive leaf levels on the 40 repeated plants, and the function best describing changes of the Mys4/Mys7 variance was determined as follows. To test whether the empirical results were better explained by monotonic or non-monotonic functions, we fitted phenomenological models whose behaviour *a priori* fitted that of the simulation results in Figure 3A. Thus a logarithmic function (y = a+b*ln(x)), and a hyperbolic-sinus function (y = d*(b/d+sinh(c+(x−a)))) were fitted to the data, plotting changes in the Mys4/Mys7 variance over leaf levels, using a maximum likelihood approach. These two functions were chosen because they could correspond to different possible patterns of changes of the viral population bottlenecks during disease progression. The logarithmic function illustrates cases where the bottleneck size is constant. The hyperbolic-sinus function illustrates the case where bottlenecks are severe at first, then relaxed for a while, and become severe again late in infection (see text of the Results section). The AIC was used to decide which of these functions best explains the observed distribution of the variance values at successive leaf levels.

### Statistical analysis

We previously reported two methods for evaluating the size of CaMV population bottlenecks, one based on a simple analysis of changes in the variance of Mys4/Mys7 relative frequency at different leaf levels, and the other using Fst statistics [25]. The application of both methods, including a slight adjustment required for the analysis of bottlenecks at leaf level 16, to our data sets is provided in the online Text S1. As already noted when first describing these methods [25], they provide very similar estimations of the bottleneck sizes, and we used only results obtained with the former in the Results section.

Other statistical analyses used various classical tests, which were all performed with the R (v2.11) package. The nature of the tests and their results are indicated in the text.

## Supporting Information

Text S1 **Online supporting information.** The online Text S1 contains additional detailed information on: i) full data sets corresponding to all results presented in this study, ii) the two statistical methods used to estimate the size of viral population bottlenecks at leaf entry, iii) the experiment showing the CaMV sap load and bottleneck size estimates on a single set of plants, iv) the Q-PCR conditions and primers, v) the detailed method for stylectomy and the problems encountered, and finally vi) the estimates of the CaMV load in aphids fed on artificial Parafilm membranes.
(DOC)
