# Supplementary material for: Circulating Virus Load Determines the Size of Bottlenecks in Viral Populations Progressing within a Host
Source: PLoS Pathog. 2012 Nov 1;8(11):e1003009. doi: 10.1371/journal.ppat.1003009 (PMC3486874; doi:10.1371/journal.ppat.1003009)
Supplement: Text S1 — Online supporting information. The online Text S1 contains additional detailed information on: i) full data sets corresponding to all results presented in this study, ii) the two statistical methods used to estimate the size of viral population bottlenecks at leaf entry, iii) the experiment showing the CaMV sap load and bottleneck size estimates on a single set of plants, iv) the Q-PCR conditions and primers, v) the detailed method for stylectomy and the problems encountered, and finally vi) the estimates of the CaMV load in aphids fed on artificial Parafilm membranes. (DOC) [file ppat.1003009.s001.doc]

***Text S1 : Gutierrez et al 2012***

**Manuscript Title:** Circulating Virus Load Determines the Size of Bottlenecks in Viral Populations Progressing Within a Host.

***Content***

1. About full data sets
2. Two methods of estimating the size of viral population bottlenecks at leaf entry
3. CaMV sap load and bottleneck size estimates on a single set of plants
4. Q-PCR conditions and primers
5. Detailed method for stylectomy and problems encountered
6. CaMV load in aphids fed on artificial Parafilm membranes

**1- Full data sets**

Full data sets correspond to i) Q-PCR quantification of the number of virus genome copies in 19 samples of pure phloem sap, ii) Q-PCR quantification of the number of virus genome copies in the total DNA extracts from 150 pools of 5 aphids summarized in Figure 1A & B and in Table S5, iii) Q-PCR normalized number of viral genome copies per actin gene from 4 leaf levels and 20 replicate plants (80 samples) summarized in Figure 1C, and iv) relative frequency of Mys4 CaMV variant (estimated by Q-PCR) in several leaf levels for each of four sets of plants : 50 plants x 4 sampling levels = 200 samples summarized in Figure 2A (blue); 50 plants x 3 sampling levels = 150 samples summarized in Figure 2A (red); 40 plants x 6 leaf levels = 240 samples summarized in Figure 3; 20 plants x 4 sampling levels = 80 samples summarized in Figure S1. Tables corresponding to either of these data sets are available upon request.

***2-Two methods estimating the size of the viral population bottleneck at leaf entry***

The two statistical methods used in this study to calculate the size of the CaMV population bottlenecks at the entry of different leaf levels have been described previously in detail [1], and are here designated the “Variance” and the “Fst” methods, respectively. Both methods estimate the size of the CaMV population bottleneck (designated Nv and Nf, respectively), when the virus colonizes a new leaf, by monitoring changes in the variance of the frequency of genetic markers over time, in 50 plants infected in parallel. The confidence interval for these estimates is provided through a resampling technique. Bootstrapping over plants is implemented by drawing a sample of 50 plants randomly with replacement, and calculating the bottleneck size. This procedure is repeated 1,000 times and a distribution of Nv and Nf is constructed. The 95% confidence intervals correspond to the 2.5 and 97.5 percentiles of these distributions.

As reported and discussed earlier, these two methods yield very similar results, as confirmed in Tables S1 and S2.

***Table S1.*** Size of CaMV population bottleneck upon entry to different leaf levels.

| “Variance” method**^a^** | | | | “Fst” method**^b^** | | | |
| --- | --- | --- | --- | --- | --- | --- | --- |
| **Leaf level** | **N°5** | **N°16** | **N°21** | **Leaf level** | **N°5** | **N°16** | **N°21** |
| **Nv** | 9.0 | 127.7* | 10.8 | **Nf** | 8.8 | 120.5 | 13.9 |
| **Mean Nv** | 9.6 | 190.2 | 12.9 | **Mean Nf** | 9.1 | 185.0 | 14.0 |
| **Median Nv** | 9.3 | 59.9 | 11.2 | **Median Nf** | 9.4 | 55.2 | 15.0 |
| **2.5% CI** | 6.6 | 19.1 | 7.1 | **2.5% CI** | 6.4 | 17.9 | 9.3 |
| **97.5% CI** | 14.4 | 908.9 | 23.9 | **97.5% CI** | 14.1 | 998.8 | 27.6 |

**^a^** A statistical analysis based on the analysis of the variance of Mys4 and Mys7 frequencies at successive leaf levels has been described previously [1]. This provides an “observed” value for the bottleneck size (Nv), as well as mean and median values, and a 95% confidence interval (CI) calculated with a resampling technique also described in [1], and referred to above.

**^b^** Analysis of the same data set based on Fst statistics has also been described previously [1]. This provides an “observed” value for the bottleneck size (Nf), mean and median values, and a 95% CI calculated with the same resampling technique [1].

* for this specific number, an adjustment of the method was required as described below.

*Testing the robustness of the statistical methods with data obtained at leaf level 16*

The estimation procedures we used were based on the increase in among-replicate variance in marker frequency, which is expected when drift is entirely responsible for changes in marker frequency. When the "final populations" variance is smaller than the "source populations" variance, these procedures yield biologically aberrant negative values [1]. This variance decrease can in principle be explained by selection, e.g. in favour of a particular variant. In the dataset used to estimate the size of the CaMV population bottleneck at leaf level 16, the "final" variance is slightly smaller than the “source” population variance (among pools of leaves below leaf 16). This slight decrease in variance cannot be due to selection because we have repeatedly verified that Mys4 and Mys7 are equi-competitive, including during this particular experiment (see figure 2A and corresponding legend). A bias induced by the presence of one or a few samples with an "atypical" behavior, i.e. too high or too low relative frequency either in the “pool of leaves” or in leaf 16, seems a more likely explanation. We investigated this further using two alternative approaches.

We first used the entire dataset and proceeded with calculation of the mean and median bottleneck values, as well as confidence intervals, using the resampling technique, exactly as described earlier (1), and using both the variance and the Fst methods (Bold lines in Table S2). Second, we applied the same procedures after eliminating progressively samples with extreme Mys4 relative frequency values. It is important to note here that when a plant showing an extreme value in the "source population" was removed, its corresponding value in leaf 16 was also removed, and vice versa. These results are reported in Table S2.

The 95% confidence intervals were strikingly similar, whether the extreme samples were gradually removed from the analysis (lines 1-4 in Table S2) or not (line 0), supporting the robustness of our method. Further, the medians of the bootstrapped distributions were quite insensitive to the number of plants removed from the original dataset. A positive value of bottleneck size was obtained when eliminating only one plant showing the highest Mys4 relative frequency and one plant showing the lowest, from both the “source” and the “final” populations. This value (line 1 in table S2) was chosen as the observed value for bottleneck size at entry of leaf level 16 (Figure 2 of the Results section) because we considered it was the most conservative. Indeed, because this leaf level is to be compared with others with narrower bottlenecks (leaf levels 5 and 21), we considered that the bottleneck value associated with the lowest limit of the confidence interval should be used preferentially.

**Table S2.** Estimating the size of CaMV population bottleneck at entry into leaf level 16

| Method | Extremes eliminated ^a^ | Number plants ^b^ | ∆var ^c^ | Number of bootstraps^d^ | Nv ^e^ | Median Nv^f^ | average Nv^f^ | 95% CI^f^ |
| --- | --- | --- | --- | --- | --- | --- | --- | --- |
|  | **0** | **48** | **-0.119** | **417** | **-210.0** | **82.4** | **300.8** | **24.2—1290..9** |
|  | 1 | 45 | 0.196 | 652 | 127.7 | 59.9 | 190.2 | 19.1—908.9 |
| Variance | 2 | 41 | 0.370 | 875 | 67.5 | 63.8 | 193.1 | 22.9—720.1 |
|  | 3 | 38 | 0.277 | 778 | 90.1 | 72.3 | 224.3 | 24.7--1016.4 |
|  | 4 | 35 | 0.298 | 804 | 84.0 | 63.2 | 748.0 | 24.7—908.7 |
|  |  |  |  |  | Nf ^e^ | median Nf^f^ | average Nf ^f^ |  |
|  | **0** | **48** | **-0.119** | **414** | **-191.7** | **73.4** | **530.9** | **21.9--1295.7** |
|  | 1 | 45 | 0.196 | 652 | 120.5 | 55.2 | 185.0 | 17.9--998.8 |
| Fst | 2 | 41 | 0.370 | 874 | 65.2 | 59.6 | 145.9 | 21.6--695.7 |
|  | 3 | 38 | 0.277 | 778 | 87.5 | 69.4 | 206.6 | 23.5--944.4 |
|  | 4 | 35 | 0.298 | 805 | 82.5 | 61.0 | 230.2 | 23.6--854.6 |

^a^ Rows in bold result from analysis of the full data set. Regular rows result from similar analyses applied to altered data sets. The number of “extremes eliminated” indicate the number of plants with the highest and the lowest Mys4 frequency values, in either the source or the final CaMV populations, that were removed from the data set.

^b^ Number of plants still included into the altered data sets

^c^ ∆var: difference between the variance in Mys4 frequency in the final and initial CaMV population (x10^2^).

^d^ Number of resampling (bootstrapping) yielding a positive value for bottleneck size.

^e^ Nv and Nf are the size of the bottleneck obtained directly from the data set, and calculated with the Variance and the Fst methods, respectively.

**^f^** Mean, median and CI of the bottleneck values result from bootstraps as described [1].

**3- CaMV sap load and bottleneck size estimates in a single set of plants**


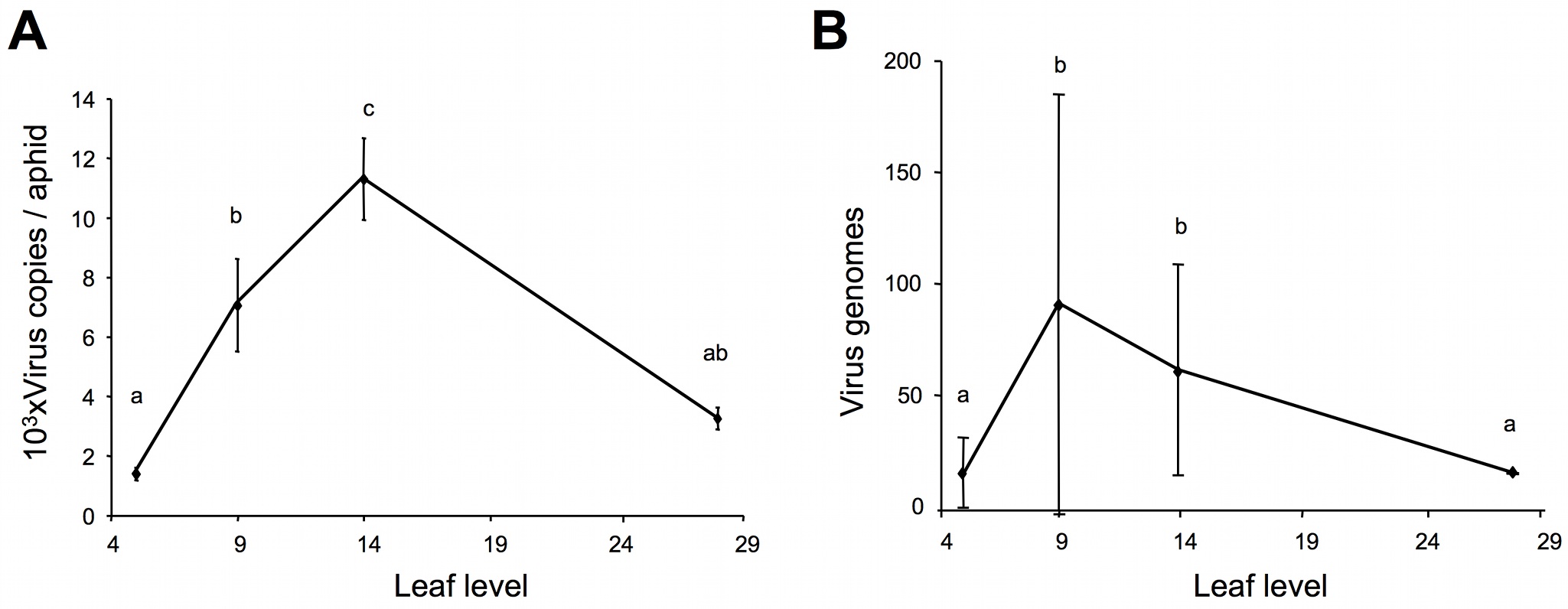


**Figure S1**. Parallel estimation of the CaMV sap loads and population sizes in different leaf levels of the same set of plants.

Each plant and long-fed-aphid DNA sample used in Figure 1 was further analyzed in order to determine the relative frequency of Mys4 and Mys7 CaMV genotypes. Thus, A and B were estimated on the same plant set and the same leaves.

(A) Virus load in aphids fed for 16 hours on the leaf levels indicated (same data as in Fig.1A). (B) Number of CaMV genomes founding the population in successive leaf levels. For each leaf level, the size of the CaMV founder population was estimated through the comparison of relative frequencies of Mys4 and Mys7, as indicated in the Materials and Methods, using the sap within aphids as the source populations and the entire corresponding leaf extracts as the final populations. Bootstraps were used to obtain 95% CI for each leaf level (see Table S3 below). A linear model showed significant differences among leaf levels (p-value < 0.0001; Tukey’s HSD, different letters mean p-values < 0.027). Vertical bars indicate standard errors.

**Table S3**: Estimates of the bottleneck sizes at the entry to different leaf-level

| Leaf level^a^ | 5 | 9 | 14 | 28 |
| --- | --- | --- | --- | --- |
| Observed value^b^ | **14,16** | **90,57** | **60,52** | **14,28** |
| mean^c^ | 60,57 | 357,27 | 204,59 | 13,68 |
| median^c^ | 14,75 | 82,58 | 65,49 | 12,79 |
| percentile 2,5^c^ | 5,37 | 31,01 | 26,45 | 8,56 |
| percentile 97,5^c^ | 204,37 | 1196,38 | 740,06 | 22,71 |
| Nb of plants^d^ | 14 | 16 | 20 | 15 |

^a^ Samples are the same as those described in Figure 1 of the main text

^b^ Calculated as described in the Materials and Methods and in [1]

^c^ Deduced from bootstrapping as described in the Materials and Methods and in [1]

^d^ At some leaf levels, some of the plants and/or aphids failed to produce a Q-PCR reliable signal

***4-PCR Protocols***

All real-time quantitative PCR reactions were carried out in a thermocycler LightCycler 480 (Roche) using the LightCycler 480 SYBR Green I Master kit, following the manufacturer’s instructions. Two microliters of the DNA extractions (either plant or aphid extracts) were used as template in the PCR reactions. The primers used are shown in Table S3 below.

The PCR program for the quantification of Mys4 and Mys7 clones was: 95ºC/10 min, 45x(95ºC/15 s, 59ºC/15 s, 72ºC/20 s), followed by a melting curve analysis.

The program for quantification of the total viral genome copies and the actin gene was identical except for the annealing temperature, which was 62ºC instead of 59ºC.

All PCR fluorescence data were analyzed using LinReg software [2]. The Mys4 frequencies in each of the samples were calculated by dividing the number of Mys4 copies by the sum of the number of copies of both Mys4 and Mys7. Plasmid dilutions containing the full CaMV genome or the *B. rapa* actin gene provided standard curves, which were used to estimate the absolute virus copy number in aphids (Figure 1A&B), or the normalized virus copy number per actin gene in plant leaves, respectively (Figure 1C).

**Table S4:** Primers used in the different PCR reactions

| **Target** | **Name** | **Sequence** |
| --- | --- | --- |
| Mys4 marker | CaMV260_1109-F | AAGAAATTGGAAATGTCCAGC |
| Mys4 marker | Mys4-R | tctacatattcctgataactcaacg |
| Mys7 marker | CaMV260_1054-F | CATCATAGCATAGATTATCAATCGAA |
| Mys7 marker | Mys7-R | agtaagtgctgtaagtataataagg |
| CaMV genome | F-cons1487 | AACAACCTCATTGAGATTGTAGGA |
| CaMV genome | R-cons1553 | TCCGAAGGGTCTTTGCTTAG |
| Actin gene | F-Act2 | GACYTBTAYGGTAACATTGTGCTC |
| Actin gene | R-actbra | TCTCTTTGCTCATACGGTCTG |

***5-Sap collection through stylectomy***

Aphids were placed on CaMV-infected turnip leaves under Electrical Penetration Graph (EPG) control, as previously described [3]. Once a clear waveform indicating sustained ingestion from sieve elements (E2>10 min) was observed, the stylet bundle was severed using a radio frequency microcautery device [4]. The radio frequency probe consisted of a parallel-tuned resonant circuit power-fed from a VHF radio frequency source, controlled by a footswitch. The probe was advanced cautiously by means of a micromanipulator until the tip of the needle gently touched the labium of the aphid, and the radio frequency source was concomitantly activated using the footswitch. The severed stylet bundle was then left inserted within the leaf tissue and the phloem sap exudates were collected using a borosilicate micro-capillary, with a tip diameter of 100 μm, filled with a small volume of silicon oil to reduce sap gelling and clogging.

Each capillary was connected to a yellow 20μl micropipette tip, and the minute volume of sap exudate was pushed out by air pressure using a P20 micropipette. The collected sap was used as template for quantifying CaMV genomes by real-time Q-PCR. Several problems compromising further use of this technique were encountered: i) the time required for an aphid to settle in the deep phloem tissues ranges from several tens of minutes to hours, and collecting numerous samples from the same leaf and from the same leaf level on many replicate plants, cannot be achieved within a day; ii) volumes collected from turnip leaves are as small as a few nanoliters, containing virus copy numbers sometimes close to the detection limit of Q-PCR; iii) borosilicate capillaries are extremely delicate and prone to breakage during connection to yellow tips and sap transfer into PCR tubes; and iv) the sap often partially or totally dries within the capillaries, and the yield of extraction with water is in such cases unsure, biasing the initially estimated volume. These difficulties prompted us to develop the alternative and more amenable procedure using whole aphids, described in the main text.

**6- CaMV load in aphids fed on artificial virus suspensions**

This experiment had two goals: (i) to validate that our protocol could reliably detect changes in the viral load among aphids having fed on suspensions with different virus concentrations, and (ii) to assess whether CaMV virions are significantly degraded within the aphid gut. Five solutions containing increasing concentrations of purified virus particles were prepared in water supplemented with 15% final sucrose. Aphids were fed during 16 hours with these solutions through artificial Parafilm membranes, and then collected for total DNA extraction. Both DNA extracts from aphids and from the feeding solutions were then analyzed by Q-PCR, and the results are summarized in Figure S2 below.


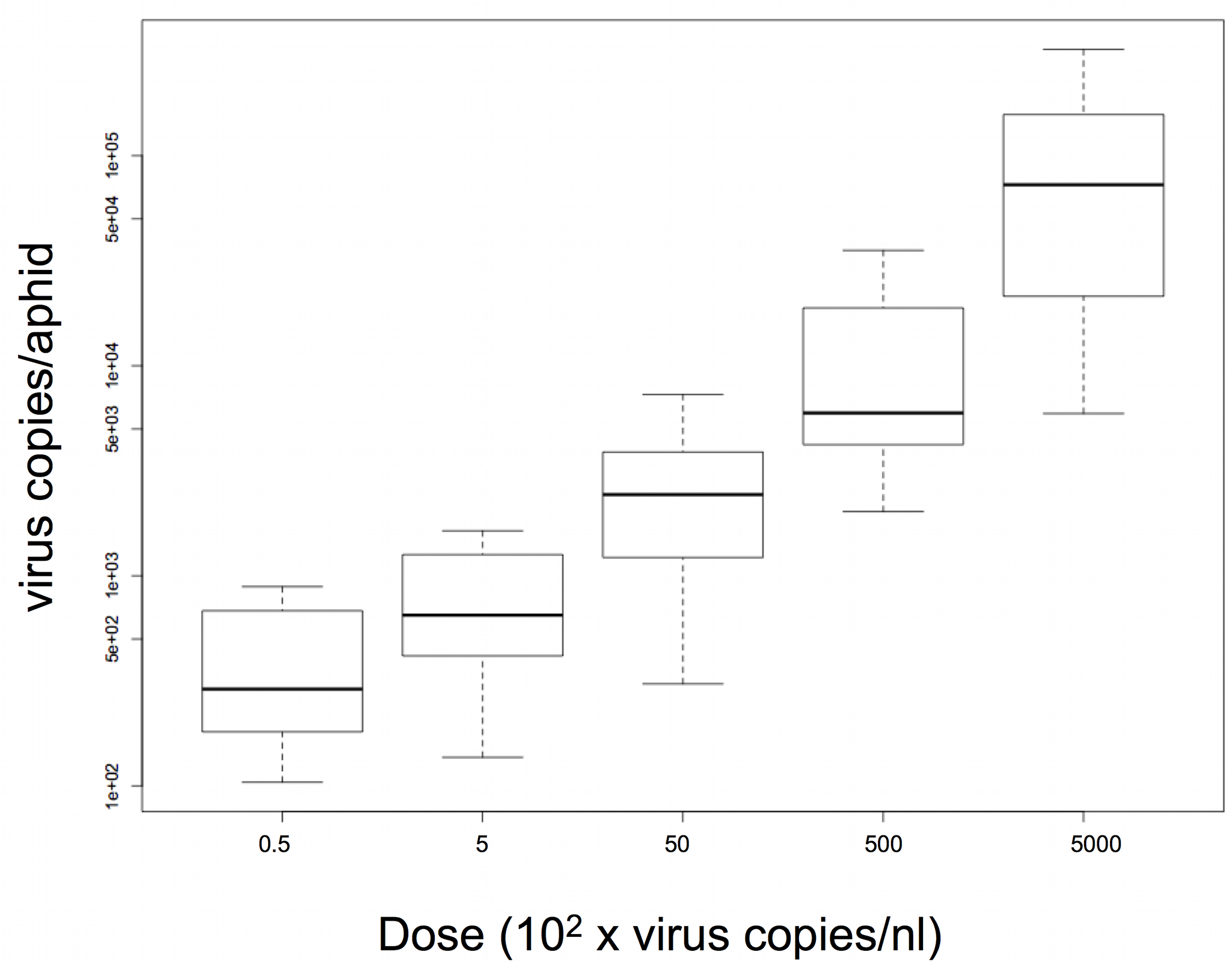


**Figure S2:** Virus load in aphids fed on artificial solutions containing increasing concentrations of CaMV virions.

Each box plot shows data distribution from three replicates. In each replicate, one hundred aphids were deposited on a membrane and allowed access to the artificial viral suspension for 16 hours. Ten pools of five aphids were then analyzed for each replicate, by extracting the total DNA and quantifying the number of viral genomes using Q-PCR. The number of viral genome copies indicated here is per aphid, in order to be directly comparable to that indicated in Figure 1. Both axes are in log-scale.

The virus load per aphid significantly increased with increasing viral concentration in the feeding-solution (linear model with membranes and concentrations as fixed effects, only the concentration effect proved significant; p-value < 0.0001). These results show that Q-PCR on aphids is a reliable relative measure of virus concentration in the ingested fluid. Nevertheless, when assuming that each aphid contains 25 nl of sap (as in the Discussion section), the calculated virus load per ingested nanoliter consistently resulted in figures about 10 times smaller than the actual viral concentration in the feeding solutions. This discrepancy could be explained in two ways: i) the assumption that aphids ingest 25 nl in average, as reported from aphids fed on plants [5], does not apply to aphids fed on artificial membranes where the ingested volume is smaller, ii) virions are partly degraded within the aphid’s digestive track. Because current literature indicates an absence of endopeptidase activity in aphid’s gut [6-8], we tested the first hypothesis by comparing aphid feeding-behavior on plant and on membrane, through Electrical Penetration Graph monitoring, as previously reported [3]. Results are summarized in Figure S3 and Table S5.

**
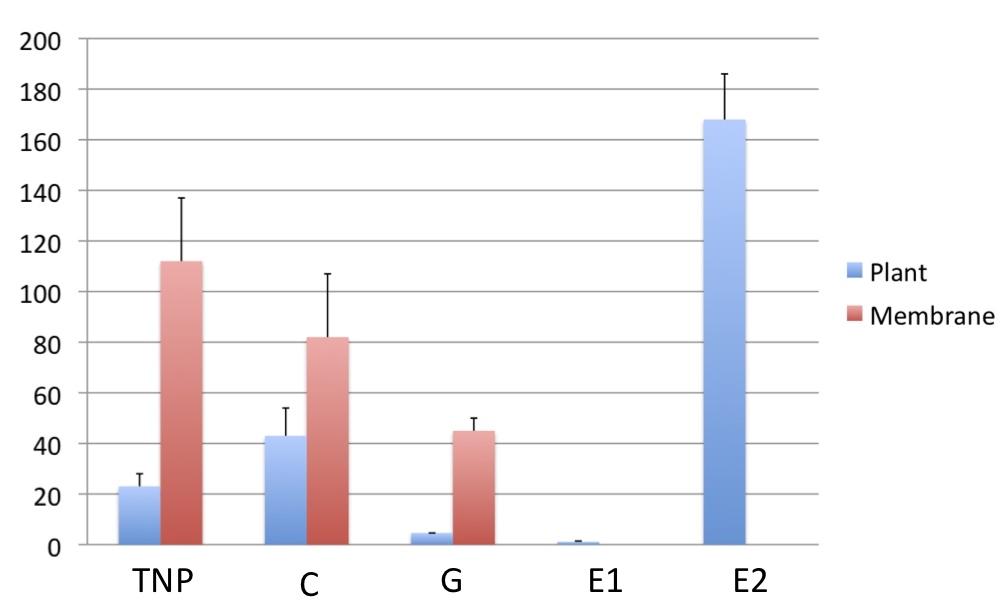
**

**Figure S3:** Comparison of main EPG components of aphid-feeding pattern on plants and on Parafilm artificial membranes.

Individual aphids were fed either on leaves (blue, a total of five aphids were monitored) or on Parafilm membranes (red, a total of 4 aphids were monitored), in a water solution containing 15% sucrose final. The total recording time was 4 hours for each aphid, and the time–scale on the left is in minutes. TNP: total non-probing time, C: pathway phase during which the aphid is probing but neither salivating nor ingesting, G: active ingestion phase during which the aphid is actively pumping the solution or the xylem sap, E1: watery saliva release, E2: passive ingestion, extremely efficient in the phloem because of the high positive pressure in sieve tubes, but impossible at atmospheric pressure of solutions on membranes. Note that aphids spend much time non-probing on membranes, and when they do probe they spend long periods in C phase where no ingestion occurs. For all phases (TNP, C, G, E1, E2) differences between plants and membranes were highly significant (Mann Whitney U test, p-values always <0.05)

**Table S5**: Main EPG components during aphid feeding on plant and Parafilm membrane.

| **EPG parameter^a^ (in min)** | **Plant-feeding** | **Membrane-feeding** |
| --- | --- | --- |
| Total non-probing time | 23 (+/- 5) | 112 (+/- 25) |
| Total time in C  (pathway phase) | 43 (+/- 11) | 82 (+/- 25) |
| Total time in G  (xylem/active ingestion) | 4.6 (+/- 0.2) | 45 (+/- 5) |
| Total time in E1  (watery salivation) | 1.1 (+/- 0.3) | 0 (+/- 0) |
| Total time in E2  (passive ingestion) | 168 (+/- 18) | 0 (+/- 0) |

^a^ All data are the same as those used to construct Figure S2

**Reference cited**

1. Monsion B, Froissart R, Michalakis Y, Blanc S (2008) Large bottleneck size in Cauliflower Mosaic Virus populations during host plant colonization. PLoS Pathog 4: e1000174.

2. Ruijter JM, Ramakers C, Hoogaars WMH, Karlen Y, Bakker O, et al. (2009) Amplification efficiency: linking baseline and bias in the analysis of quantitative PCR data. Nucleic Acids Research 37(6): e45.

3. Palacios I, Drucker M, Blanc S, Leite S, Moreno A, et al. (2002) Cauliflower mosaic virus is preferentially acquired from the phloem by its aphid vectors. J Gen Virol 83: 3163-3171.

4. Downing N, Unwin DM (1977) A new method for cutting the mouth-parts of feeding aphids, and for collecting plant sap. Physiological Entomology 2: 275-277.

5. Wright JP, Fisher DB, Mittler TE (1985) Measurement of the aphid feeding rates on artificial diets using 3H-inulin. Entomol Exp Appl 37: 9-11.

6. Cristofoletti PT, de Sousa FA, Rahbe Y, Terra WR (2006) Characterization of a membrane-bound aminopeptidase purified from Acyrthosiphon pisum midgut cells. A major binding site for toxic mannose lectins. Febs J 273: 5574-5588.

7. Pyati P, Bandani AR, Fitches E, Gatehouse JA (2011) Protein digestion in cereal aphids (Sitobion avenae) as a target for plant defence by endogenous proteinase inhibitors. J Insect Physiol 57: 881-891.

8. Rahbe Y, Sauvion N, Febvay G, Peumans WJ, Gatehouse AMR (1995) Toxicity of Lectins and Processing of Ingested Proteins in the Pea Aphid Acyrthosiphon-Pisum. Entomologia Experimentalis Et Applicata 76: 143-155.
